# Evaluation of placental oxygenation by near-infrared spectroscopy in relation to ultrasound maturation grade in physiological term pregnancies

**DOI:** 10.1515/med-2023-0843

**Published:** 2023-11-07

**Authors:** Marinela Bakotin Jakovac, Andrea Etrusco, Mislav Mikuš, Damir Roje, Jelena Marusic, Ivan Palada, Indira Kosovic, Nadja Aracic, Martina Sunj, Antonio Simone Laganà, Vito Chiantera, Zeljko Dujic

**Affiliations:** Hormona Polyclinic, Kranjceviceva 45, 21000 Split, Croatia; Department of Health Promotion, Mother and Child Care, Internal Medicine and Medical Specialties (PROMISE), University of Palermo, 90133 Palermo, Italy; Unit of Obstetrics and Gynecology, “Paolo Giaccone” Hospital, 90127 Palermo, Italy; Department of Obstetrics and Gynecology, Clinical Hospital Center, 10000 Zagreb, Croatia; Department of Health Studies, University of Split, 21000 Split, Croatia; Roda Polyclinic, 21000 Split, Croatia; Cito Polyclinic, 21000 Split, Croatia; Unit of Gynecologic Oncology, National Cancer Institute – IRCCS – Fondazione “G. Pascale”, 80131 Naples, Italy; University of Split School of Medicine, 21000 Split, Croatia; Department of Obstetrics and Gynecology, University Hospital Center, 21000 Split, Croatia

**Keywords:** near-infrared spectroscopy, placenta, placental oxygenation, tissue oxygenation index, ultrasound

## Abstract

A prospective observational study (ClinicalTrial ID: NCT05771415) was conducted to compare placental oxygenation in low-risk, uncomplicated term pregnancies measured by near-infrared spectroscopy (NIRS) in relation to the placental maturity grade determined by ultrasound assessment according to the Grannum scale. We included 34 pregnancies divided into two groups according to placental maturation. For each pregnancy, measurements were taken at the site above the central part of the placenta (test) and at the site outside of the placenta on the lower abdomen (control). Student’s *t*-test was used to compare tissue oxygenation index (TOI) values among the study groups. The normality of distribution was proven by the Kolmogorov‒Smirnov test. In women with low placental maturity grade, the mean TOI value above the placenta was 70.38 ± 3.72, which was lower than the respective value in women with high placental maturity grade (77.99 ± 3.71; *p* < 0.001). The TOI values above the placenta and the control site were significantly different in both groups (70.38 ± 3.72 vs 67.83 ± 3.21 and 77.99 ± 3.71 vs 69.41 ± 3.93; *p* < 0.001). The results offer a new perspective on placental function based on specific non-invasive real-time oxygenation measurements. Unfortunately, and because of technical limitations, NIRS cannot yet be implemented as a routine clinical tool.

## 1 Introduction

Uteroplacental circulation is the site of the closest metabolic contact between the mother and the fetus, whereby oxygen transport to the fetus is of utmost importance [1]. The following direct and indirect methods are currently used to monitor placental function: cardiotocography (CTG), ultrasonography (US) with Doppler measurements, biophysical profiling, ST segment analysis of fetal electrocardiogram (STAN), amnioscopy, pulse oximetry, and pH-metry [2], aimed at identifying fetal hypoxia and offering prompt and proper treatment. Nevertheless, despite available clinical guidelines based on their combinations aimed to optimize risk pregnancy surveillance, sometimes adverse pregnancy outcomes occur [3]. Histopathologic evaluation is currently the only definitive analysis that can be performed to assess the placental function [4]; unfortunately, it can only be performed postpartum and not during pregnancy.

Therefore, new methods to follow-up placental respiratory function are needed. Research has been focused on 3D and 4D US [5], functional magnetic resonance imaging [6], and near-infrared spectroscopy (NIRS) [7,8]. The latter is the latest and least studied method, which offers the possibility of non-invasive determination of tissue oxygenation by continuous measurement of the amount of oxygen available at the microcirculation level [7,9,10,11,12]. It also represents the first method that could be used as follow-up, where respiratory factors are measured within the placenta [9,10,11,12].

Ultrasound assessment of placental maturity relies on the characteristics of hyperechoic signals [13]. Based on their amount, magnitude, and pattern, a placenta is classified into four groups according to the Grannum scale [14].

The role of placental maturity grade depends on gestational age. Most authors agree that higher maturity grades in the second trimester are associated with a higher probability of subsequent development of preeclampsia and/or intrauterine growth retardation (IUGR) [15]. The possible correlation of the same finding in the third trimester has not yet been confirmed. Current methods of fetal or placental function in term pregnancy often fail to indicate any association with adverse perinatal outcomes. In addition, the potential subclinical effect on placental respiratory function has never been established or rejected [13,16].

Considering these elements, the aim of this study was to compare low-risk placental oxygenation in uncomplicated term pregnancies measured by NIRS in relation to the degree of placental maturity determined by US assessment according to the Grannum scale.

## 2 Methods

### 2.1 Patients

We conducted our research (ClinicalTrial ID: NCT05771415) by collecting data from October 1, 2019, to February 28, 2021. The study included only pregnant women with singleton low-risk, uncomplicated, term pregnancies (37^+0^ to 41^+6^ weeks of gestation), anterior placental localization and an abdominal wall of less than 3 cm thickness, and uncomplicated vaginal deliveries (no adverse maternal–fetal outcomes). US and Doppler studies, as well as CTG and laboratory findings, were normal in all women included in this study.

The women were divided into two groups:

- Group 1: Grannum placental maturity grade 0 and 1 (low maturity level);
- Group 2: Grannum placental maturity grades 2 and 3 (high maturity level) [14].

### 2.2 Placental oxygenation measurement

Assessment of the degree of placental maturity was made for all patients between 37^+0^ and 41^+6^ weeks of pregnancy. After 30 min of rest, all patients who participated in the study were initially examined by ultrasound by an experienced obstetrician to assess the placental maturity and assigned to one of two groups. After the ultrasounds, placental oxygenation was measured. Placental oxygenation was continuously determined by the NIRS method on a Hamamatsu NIRO-200 device (*Hamamatsu Photonics KK, Tokyo, Japan*). One 40 mm probe was placed at the control site on the lower abdomen, out of the placenta, in a medial line 3 cm above the symphysis. The other probe, of the same size, was positioned at the same place as indicated by previous ultrasound of the placenta (the site corresponded in most cases to 2–3 cm above the umbilicus along the midline). Thus, both ultrasound and NIRS measurements were made in a very short time for each woman. This allowed us to obtain reliable data on the degree of maturity of the placenta and how much this degree of maturity affects disturbed oxygenation in that moment of evaluation.

The NIRS device was used for continuous measurement of changes in the absorption of near-infrared rays by oxygenated and deoxygenated hemoglobin, from which the level of placental oxygen saturation was calculated. The digital signal of the overall NIRS measurement was continuously recorded and stored on a PC using an analog-digital transducer (*Power Lab 16S data acquisition system, ADInstruments, Castle Hill, Australia*). The mean value of placental oxygenation measured by NIRS during a 60-s interval was then calculated from the records using Power Lab software (*ADInstruments, Castle Hill, Australia*). The 60-s interval was chosen as the first in a series on a continuing record within a segment free from artifacts.

The software automatically calculated the mean levels of oxygenated and deoxygenated hemoglobin for the respective interval and, from these levels, the values of the tissue oxygenation index (TOI) were determined.

The TOI was calculated by the following formula [17]:

\[\frac{100\left\times {\mathrm{Oxyhemoglobin}}\left({{\mathrm{HbO}}}_{2})\hspace{1em}}{{\mathrm{Oxyhemoglobin}}\hspace{1em}({{\mathrm{HbO}}}_{2})\left+{\mathrm{Deoxyhemoglobin}}\left({\mathrm{HHB}})}.]\]

Statistica 7.0 software (*StatSoft, Inc., Tulsa, USA*) was used for statistical analysis. The results are expressed as the mean ± standard deviation (SD). Student’s *t*-test was used to compare TOI values among the study groups. The normality of distribution was confirmed by the Kolmogorov‒Smirnov test. In all analyses, the level of statistical significance was set at *p* < 0.05.


**Ethics approval and consent to participate:** The study was approved by the Ethics Committee of the University Hospital of Split (Reference: 2181-147-09-01/01-M. J; date of approval 17 March 2019).The design, analysis, interpretation of data, drafting, and revisions are in compliance with the Helsinki Declaration, the Committee on Publication Ethics guidelines, and the Strengthening the Reporting of Observational Studies in Epidemiology Statement [18], validated by the Enhancing the Quality and Transparency of Health Research Network. The study was not advertised, and no remuneration was offered to encourage patients to give consent for the collection and analysis of their data. Each patient enrolled in this study was informed about the aims and procedures and signed an informed consent form to allow data collection for research purposes.

## 3 Results

The study included 34 pregnant women, 10 of whom had a low placental maturity grade (group 1, Grannum 0–1) and 24 of whom had a high placental maturity grade (group 2, Grannum 2–3) [14].

There were no statistically significant differences between the two groups according to age (*p* = 0.920), height (*p* = 0.721), weight (*p* = 0.621), or body mass index (BMI) at the time of measurement (*p* = 0.718).

All deliveries occurred in the 40th week on average (no significant differences between the two groups; *p* = 0.355), with no statistically significant differences according to neonatal birth weight (*p* = 0.124), birth length (*p* = 0.703), or ponderal index (*p* = 0.234). Measurement by the NIRS method performed in the 40th week of gestation in both groups showed that the abdominal wall thickness ranged from 0.9 to 2.7 cm, with no significant difference (*p* = 0.28) between the groups for this parameter (Table 1).

**Table 1:** Baseline characteristics in women with low placental maturity grade (Grannum 0–1) and high placental maturity grade (Grannum 2–3)

	Low placental maturity grade (Grannum 0–1), *n* = 10	High placental maturity grade (Grannum 2–3), *n* = 24	p*
Maternal age (years)	30.1 ± 4.6	30.2 ± 4.5	0.920
Maternal height (cm)	168 ± 4.75	170 ± 4.73	0.721
Maternal weight (kg)	81.6 ± 10.5	84.3 ± 1 0.1	0.610
BMI (kg/m^2^) at the time of measurement (weeks)	28.6 ± 3.44	28.8 ± 3.06	0.718
Gestational age at the time of measurement (weeks)	39.7 ± 1.15	39.2 ± 0.97	0.351
Maternal abdominal wall thickness at the time of measurement (cm)	1.37 ± 0.39	1.42 ± 0.49	0.28
Gestational age at delivery (weeks)	39.9 ± 0.99	39.5 ± 0.97	0.355
Birth weight (g)	3658 ± 187	3718 ± 478	0.124
Birth length (cm)	51.7 ± 1.59	51.4 ± 1.95	0.703
Ponderal index (g/cm^3^)	2.66 ± 0.15	2.73 ± 0.22	0.234

*Student’s *t*-test.

The values of TOI measured over the placenta and at the control site yielded statistically significant differences in the group with low maturity grade (70.38 ± 3.72 vs 67.83 ± 3.21; *t* = 6.74; *p* < 0.001), as well as in the group with high maturity grade (77.99 ± 3.71 vs 69.41 ± 3.93; *t* = 9.38; *p* < 0.001). Measurements above the placenta showed significant differences between the groups.

In group 2, which had a high placental maturity grade, the TOI was significantly higher than that in group 1, which had a low placental maturity grade (77.99 ± 3.71 vs 70.38 ± 3.72; *t* = 6.09; *p* = 0.228). No significant differences were found between the groups in the TOI values measured at control sites (69.3 ± 3.93 vs 67.83 ± 3.21; *t* = 1.224; *p* = 0.228) (Table 2).

**Table 2:** TOI values in women with low placental maturity grade (Grannum 0–1) and high placental maturity grade (Grannum 2–3)

	Low placental maturity grade (Grannum 0–1), *n* = 10	High placental maturity grade (Grannum 2–3), *n* = 24	p*
TOI measured over placenta	70.38 ± 3.72	77.99 ± 3.71	<0.001
TOI measured at control site	67.83 ± 3.21	69.41 ± 3.93	0.228
p*	<0.001	<0.001	

*Student’s *t*-test.

## 4 Discussion

In 1977, Jöbsis introduced the concept of NIRS for measuring oxygenation in various intact organs in the fields of neurology and cardiology [10]. Initial studies in perinatology were conducted on an animal model 25 years later: Choe et al. demonstrated the use of NIRS, a non-invasive method for measuring tissue oxygenation, as an effective alternative to traditional direct invasive methods; this study was conducted on five sheep and produced results comparable to those obtained from measuring arterial and venous blood oxygenation [11]. Other robust data from a mouse model studied the transfer of indocyanine green (ICG) across the placenta and its distribution within fetal tissues, as well as the effects of concomitant drugs on fetal exposure, using NIRS [19]. The distribution of ICG in the mouse fetus may be enhanced when used concomitantly with organic anion transporting polypeptide (OATP) or P-glycoprotein inhibitors. The enhanced distribution in individual fetal tissues is probably related to the increased transplacental transfer of ICG. Although these are data from preclinical studies, it is therefore possible to hypothesize that, similar to what occurs in the animal model after the administration of OATP or P-glycoprotein inhibitors, placental NIRS results may be influenced by maternal drug use. In 2005, Kakogawa et al. pioneered the use of NIRS in the assessment of placental respiratory function in human medicine [12]. Japanese authors used the NIRS method to compare the oxygenation of the placenta in both normal and pathologic pregnancies [9,12,20,21,22]. Hasegawa et al. conducted the largest study on the topic, including a sample of 326 pregnant women [22], and found increased TOI in pregnancies with preeclampsia, intrauterine growth restriction (IUGR), and gestational diabetes. The measurements were taken at various gestational ages, and in many cases, the thickness of the maternal abdominal wall was more than 3 cm [9,12,20,21,22,23,24,25].

Kawamura et al. [21] reported on TOI values recorded in a healthy control group that corresponded to those found in our lower placental maturity grade group (70.2 ± 0.4 vs 70.37 ± 3.72). In their study, TOI values recorded in the IUGR group corresponded to those measured in our group of women with higher placental maturity grade (78.6 ± 1.6 vs 77.9 ± 3.7).

Suzuki et al. [24] demonstrated a correlation of the TOI value calculated from the NIRS measurement of placental oxygenation with the postpartum histopathologic placental finding. In a sample of 19 placentas in which chorangiosis was histopathologically verified postpartum, TOI was elevated irrespective of IUGR condition.

Hasegawa et al. [22] reported a decrease in TOI with gestational age, although they did not propose a robust explanation for this phenomenon. In this scenario, the outcome of our study may address the inclusion of placental maturity grade (Grannum) in future study designs. Nguyen et al. [26] introduced a new NIRS device with six different source–detector distances from 10–60 mm, which is able to measure the optical properties of the front placental tissue up to 25 mm deep. This flexible device eliminates the impact of scattering effects on placental oxygenation levels [26,27].

Recently, new forms of NIRS devices have been created. These devices combine optical instruments with ultrasound imaging in one probe head, providing a multimodal combination of frequency domain diffuse optical spectroscopy/ultrasound. This integration enables the combination of anatomical ultrasound information about tissue layer morphology with functional hemodynamic information about deeper tissues. The anatomical information allows for tissue-specific, layered image reconstruction, which separates the hemodynamic properties and responses of deep tissues such as the placenta from those of the overlying layers [28].

Overall, the results of the present study showed that the level of placental oxygenation, as measured by NIRS, is higher in women with a high US-assessed Grannum placental maturity grade. Nevertheless, we acknowledge that measurement results obtained by NIRS can be neither uniform nor simple to interpret, especially in a clinical context. Indeed, from the probe toward distal areas, the space of measurement as determined by the protocol includes the maternal abdominal wall, anterior uterine wall, placenta, and, to a minor extent, amniotic fluid.

To minimize the potential bias, we used a threshold of maternal abdominal wall thickness less than 3 cm as one of the main inclusion criteria. The control site measurement was also performed for the same reason and showed no significant differences between the groups, reducing the possibility of bias caused by obesity and composition of the maternal abdominal wall and anterior uterine wall.

Furthermore, the measurement area was predominantly placental tissue, which is mostly (60%) composed of trophoblast and to a lesser extent (40%) of intervillous space (IVS) filled with approximately 200 mL of maternal blood [29].

The level of oxygenation as measured by NIRS was expressed as TOI, which represents the proportion of oxyhemoglobin (HbO_2_) to total hemoglobin within the measured area. The total placental HbO_2_ level reflects the HbO_2_ concentration in the trophoblast and IVS as its basic anatomic components. IVS consists exclusively of maternal blood, whereas the proportion of fetal blood components and thus the HbO_2_ concentration in the trophoblast is far lower [30].

Therefore, the TOI value is largely dependent on the composition of the IVS and the level of maternal blood oxygenation, with only a minor contribution from fetal parameters. As the ratio of trophoblast to IVS volume is typically constant, the measured TOI value can mostly be considered a surrogate parameter of IVS oxygenation [31]. Nevertheless, the minor effect of the trophoblast component should not be underestimated.

Generally, a higher TOI means more HbO_2_ in the IVS, reflecting a lower placental transport oxygen capacity through the trophoblast layer into fetal circulation. To a further extent, TOI can also be described as “relative oxygen accumulation” in the IVS.

To date, the value of placental TOI has been interpreted exclusively in the context of IVS [9,20,21,22]. In the current scenario, the well-defined gestational age range and strict abdominal wall thickness criteria (less than 3 cm) in our protocol helped ensure the objectivity of our results.

In our study, the mean TOI value recorded over the placenta was 11.5% higher in the group with high placental maturity grade and only 3.5% higher in the group with low placental maturity grade compared to the control site. Of note, the TOI values measured at control sites were nearly the same in both groups (67.83 ± 3.21 vs 69 ± 3.93). These findings might indicate that a higher placental maturity grade affects placental respiratory dynamics, but at a subclinical level.

Previous studies have not addressed the impact of placental maturity on placental oxygenation, and the groups have not been standardized based on this criterion. As a result, no firm conclusions can yet be drawn.

Our findings, however, show variations in placental oxygenation in the third trimester of normal pregnancies based on the level of placental maturity, suggesting that more advanced placental maturation may have a subclinical impact on respiratory function.

Although the present study does not intend to give placenta ultrasound maturity grading any further clinical significance, these results represent a new challenge for further investigations on placental oxygenation. Furthermore, data from several authors seem to confirm the theory that a more mature placenta is associated with worse pregnancy outcomes [32,33,34,35]. Indeed, the increased level of histologically visible calcification, which is one of the key criteria for determining the degree of placental maturity according to Granum, also leads to worse perinatal outcomes [36].

To date, NIRS offers a new and direct option for intraplacental oxygen measurement [37,38]. Although the method has the benefits of being safe and relatively objective, its drawbacks should also be acknowledged. Specifically, the measurement site represents only a portion of the overall placental volume; the result is greatly influenced by HbO_2_ concentration in the IVS (maternal compartment) and to a smaller extent by the trophoblast (fetal compartment). Moreover, the measurement cannot be taken if the site is different from the anterior part of the placenta and if the distance between the probe and the placenta exceeds 4.5 cm.

The information acquired by this method of placental oxygenation measurement can only be considered a step forward in the understanding of placental function, particularly its respiratory component. Although NIRS is still far from being implemented as a routine clinical tool, it may offer a new perspective through non-invasive and real-time oxygenation measurement within the placenta. Overall, we acknowledge that the generalizability of our results is limited by the low number of participants enrolled and the inclusion of only physiologic pregnancies at term. Future studies should confirm these results in larger cohorts and compare physiologic and pathologic pregnancies at different gestational ages to clarify the potential role and feasibility of NIRS in clinical practice.
